# Analysis of *vkorc1 *polymorphisms in Norway rats using the roof rat as outgroup

**DOI:** 10.1186/1471-2156-11-43

**Published:** 2010-05-24

**Authors:** Juan C Díaz, Ying Song, Anthony Moore, Jeff N Borchert, Michael H Kohn

**Affiliations:** 1 Department of Ecology and Evolutionary Biology, Rice University, 6100 Main Street, MS 170, Houston, Texas 77005, USA; 2 Centers for Disease Control and Prevention Division of Vector-Borne Infectious Diseases, Bacterial Diseases Branch, 3150 Rampart Road, Fort Collins, CO 80522, USA

## Abstract

**Background:**

Certain mutations in the vitamin K epoxide reductase subcomponent 1 gene (*vkorc1*) mediate rodent resistance to warfarin and other anticoagulants. Testing for resistance often involves analysis of the *vkorc1*. However, a genetic test for the roof rat (*Rattus rattus*) has yet to be developed. Moreover, an available roof rat *vkorc1 *sequence would enable species identification based on *vkorc1 *sequence and the evaluation of natural selection on particular *vkorc1 *polymorphisms in the Norway rat (*R. norvegicus*).

**Results:**

We report the coding sequence, introns and 5' and 3' termini for the *vkorc1 *gene of roof rats (*R. r. alexandrinus *and *R. r. frugivorus*) from Uganda, Africa. Newly designed PCR primers now enable genetic testing of the roof rat and Norway rat. Only synonymous and noncoding polymorphisms were found in roof rats from Uganda. Both nominal subspecies of roof rats were indistinguishable from each other but were distinct from *R. losea *and *R. flavipectus*; however, the roof rat also shares at least three coding sequence polymorphisms with *R. losea *and *R. flavipectus*. Many of recently published *vkorc1 *synonymous and non-synonymous single nucleotide polymorphisms (SNPs) in Norway rats are likely SNPs from roof rats and/or other *Rattus *species. Tests applied to presumably genuine Norway rat *vkorc1 *SNPs are consistent with a role for selection in two populations carrying the derived Phe63Cys and Tyr139Cys mutations.

**Conclusion:**

Geographic mapping of *vkorc1 *SNPs in roof rats should be facilitated by our report. Our assay should be applicable to most species of *Rattus*, which are intermediate in genetic distance from roof and Norway rats. *Vkorc1*-mediated resistance due to non-synonymous coding SNPs is not segregating in roof rats from Uganda. By using the roof rat sequence as a reference *vkorc1*, SNPs now can be assigned to the correct rat species with more confidence. Sampling designs and genotyping strategies employed so far have helped detect candidate mutations underlying *vkorc1*-mediated resistance, but generally provided unsuitable data to test for selection. We propose that our understanding of *vkorc1*-mediated evolution of resistance in rodents would benefit from the adoption of sampling and genotyping designs that enable tests for selection on *vkorc1*.

## Background

The roof rat (*Rattus rattus*) is one of the most abundant and geographically widespread mammals [1]. It has prominently influenced human history by acting as a carrier of disease, most prominently the plague, which remains a concern for public health particularly in Asia and Africa. The roof rat and numerous closely related species are important agricultural pests. Damage to structures in urban areas and devastating effects on endemic fauna on islands or otherwise endemic habitats are also areas of great concern. Pest management of roof rat populations is a global task [1].

Anticoagulant rodenticides target vitamin K epoxide reductase (VKOR) [2], an essential enzyme for the vitamin K cycle (c.f. [3] for review and additional references). Inhibition of VKOR by the rodenticides prevents production of functional blood coagulation factors, such that susceptible rodents that consume the bait succumb to lethal internal hemorrhage (c.f. [4] for review and additional references). A warfarin-sensitive subcomponent of VKOR is encoded by the vitamin K epoxide reductase complex subunit 1 (*vkorc1*) gene [5,6].

*In vivo *and *in vitro *studies showed that certain single-nucleotide polymorphisms (SNPs) in the coding region of the *vkorc1 *confer resistance in Norway rats (*R. norvegicus*), mice (*Mus musculus *spp.) [7] and *R. losea *[8]. Moreover, SNPs in both the coding and 5' non-coding regions of the gene are associated with warfarin (a.k.a. coumadin) responsiveness in humans [9]. However, the physiological response to anticoagulants can also be affected or modified by other genes, including cytochrome P450 genes in roof rats from Japan [10] and in humans (*cytochrome P450 2C9*) (e.g. [9]). Nevertheless, while other genes might modify physiological tolerance levels and possibly resistance, this phenomenon is currently investigated in rodents mainly with respect to SNPs in the *vkorc1 *gene [11]).

Anticoagulant rodenticides remain the tool of choice to control infestations by many species of rodents [12]. However, unlike in the Norway rat and the mouse, it is not known whether roof rat populations carry SNPs in *vkorc1 *that might render them tolerant or resistant to anticoagulant rodenticides. As for Norway rats and house mice, variation in tolerance towards rodenticides has been observed for the roof rat [e.g. [10,13]]. Geographic mapping of *vkorc1 *SNPs in this species across the globe is required, especially in areas where the roof rat is considered a nuisance species. Such genetic testing could identify individuals or populations that merit more conclusive but also more labor- and cost-intensive *in vitro *and/or *in vivo *laboratory testing for resistance [7,11]. The detection of *vkorc1*-mediated resistance in roof rats would also be interesting from an evolutionary perspective, because it would provide additional evidence for convergent evolution of *vkorc1*-mediated poison resistance in multiple rodent species. However, while the *vkorc1 *genes of the Norway rat and house mouse have been sequenced as part of genome sequencing projects and population sequencing surveys, the *vkorc1 *sequence of the roof rat remains unknown. Consequently, there is no genetic toolkit presently available that can be used to conduct genetic testing of the roof rat.

The identification of many *vkorc1 *SNPs in Norway rats has revealed direct links for some of these to resistance in this species, most notably at amino acid position 139 of the protein [6,7,11]. When mutated, it was shown that this position confers resistance *in vivo *and *in vitro *[7,14]. However, as evidenced from alignments among *Drosophila*, fish and mammals, the *vkorc1 *gene is highly conserved. Thus, the large number of SNPs in the *vkorc1 *gene of the Norway rat has motivated biological interpretations, including the presence of mutational hotspots and selection [7].

Population genetic theory predicts that most SNPs in a finite sample of sequences are detrimental or neutral [15]. The finding of numerous *vkorc1 *SNPs in a finite sample of Norway rats suggests that these must be frequent or else they would not have been found in the small samples analyzed to date for the Norway rat [7]. This has prompted speculation that these SNPs must be adaptive, possibly in the context of fifty years of warfarin selection [7]. However, we suggest that, in layman's terms, the degree of polymorphism reported for the Norway rat seems biologically implausibly high. This prompted us to reevaluate *vkorc1 *SNPs that have been published for the Norway rat using the outgroup sequence of *vkorc1 *from the roof rat obtained as part of our study reported here.

Except for a few studies based on microsatellite data [16,17] no other population genetic tests for selection at the *vkorc1 *have been conducted. The published SNP data for the Norway rat should be subjected to rigorous analyses, however such analyses require appropriate outgroup sequences to determine which SNPs are ancestral and which ones are derived. The latter group more likely represents SNPs that mediate resistance to rodenticides. The *vkorc1 *sequence of the roof rat could provide such a needed outgroup perspective. Moreover, the published SNP data have yet to be explored in terms of the plausibility of selection on the *vkorc1 *arising from widespread use of warfarin and other anticoagulant poisons. Such explorations of currently published *vkorc1 *SNPs might illustrate the need to apply different sampling strategies from those currently used to enable population genetic tests for selection. For example, published *vkorc1 *SNP data generally appear to have been collected following non-random sampling designs of rats, which, essentially, focused on strains or populations where resistance was *a priori *known or suspected to be present, and thus, the likelihood of detecting non-synonymous candidate mutations in *vkorc1 *underlying resistance was high. Moreover, preference was given to sampling designs that included few individuals each but from many locations worldwide. Thus, in general, published *vkorc1 *SNPs are from non-random samples of few individuals.

Here we report the complete genomic sequence of the *vkorc1 *gene from the roof rat. Our study is set within the context of ecological and parasitological surveys of roof rats and other rodents in the West Nile region of Northwest Uganda, where the bubonic, septicemic and pneumonic forms of the plague still occur [18]. The management of non-native roof rat populations and other native rodent species that can carry and/or transmit diseases is considered within this context, as is the need for genetic testing of roof rats for the presence of *vkorc1 *SNPs that might interfere with rodent control.

We provide primers for PCR amplification and DNA sequencing, namely a genetic testing strategy for the presence of SNPs in *vkorc1 *that is applicable to roof rats. However, we show that the strategy can also be applied to the Norway rat, thereby eliminating the need to apply species-specific strategies in areas where both species are found. Furthermore, we designed the approach so it can be tested on the many rat pest species that are intermediate in genetic distance to the roof and Norway rats. We present polymorphism data on the two putative subspecies *R. r. alexandrinus *and *R. r. frugivorus*, and show that the *vkorc1 *sequence of *R. rattus *is distinct from the partial sequences reported for *R. flavipectus *and *R. losea*. We illustrate the value of the roof rat *vkorc1 *sequence by reanalyzing published SNP data for the Norway rat, both in terms of species identification and the possibility of selection on the gene.

## Results and Discussion

### The *vkorc1 *sequence of the roof rat

#### Amplification of *vkorc1 *in rats

**Figure 1:**
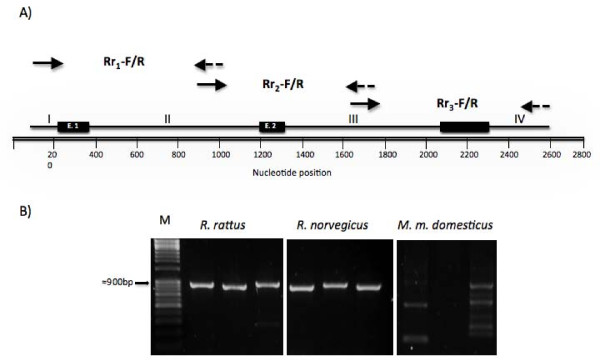
**Overview of *vkorc1 *sequence of *R. rattus *and PCR amplification strategy**. (A) Depicted are the *vkorc1 *gene exons 1-3 (E.1-E.3), 5' and 3' termini (I, IV) and introns (II, III). Shown are the relative positions of the primers Rr_1_-F and -R [5'-TAGCTGTCACGCCTAAGAA-3'; 5'-GCAAATAAGTGCCTGCTGCC-3'], Rr_2_-F and -R [5'-ACTTTCGGGAGCTGATTCTC-3'; 5'-CAGACTGATTTTATGTAATG-3'] and Rr_3_-F and -R [5'-CAGGGTTTCTCTGTGTAAC-3'; 5'-CAGACTTGACCAACATAGAA-3'] that cover *vkorc1*. **(B) **Specific PCR amplicons representing three segments covering *vkorc1 *resolved by 1% agarose gel electrophoresis. From left to right: M-1 kb size ladder (Fisher Scientific International, Pittsburgh, PA, USA), PCR products obtained using Rr_1_-F/R, Rr_2_-F/R and Rr_3_-F/R primer pairs for *R. rattus*, *R. norvegicus*, and *M. m. domesticus*.

The primer combinations Rr_1_-F/R, Rr_2_-F/R and Rr_3_-F/R amplified the 5' and 3' noncoding regions (I and IV), both introns (II and III) and all three exons (E.1-E.3) for the roof rat (Figure 1A) and the Norway rat, but the primers are unsuitable to amplify corresponding regions from mouse DNA (e.g. here *M. m. domesticus *collected in Western Germany) under standard conditions (Figure 1B). The primer combination Rr_1_-F/R, Rr_2_-F/R and Rr_3_-F/R should be useful for screening roof rat populations for *vkorc1 *polymorphisms.

We designed the genetic assay in order to provide a test that is applicable to the many other pest species of *Rattus *(e.g., *R. losea *and *R. exulans*) that are intermediate in genetic distance between the roof and Norway rats, which diverged ~2.8 million years ago [19,20]. This would enable the application of one assay even in populations where numerous *Rattus *species occur together, in particular the many locations where the cosmopolitan species (roof rat and Norway rat) are sympatric. However, as we explore below, this raises issues regarding correct assignment of SNPs in the *vkorc1 *that emerge from genetic testing in areas where the roof and Norway rats are both found, or in other assemblages of rat species [21].

#### Uniqueness of the roof rat sequence

Inspection of the *vkorc1 *coding sequence showed one fixed synonymous substitution (T280C) between the roof rat and *R. losea*/*R. flavipectus*; the remaining polymorphisms in the roof rat are shared among the three species (Additional file 1 for protein coding sequence data and Additional file 2 for 5'UTR/intron sequence data). This is testament to their recent common ancestry (~0.45 million years ago, [19]), and/or that the incipient species resulting from the recent if not ongoing radiation of the *R. rattus *species complex (a group of dozens morphologically highly similar and often indistinguishable species including *R. argiventer, R. tiomanicus, R. sikkimensis*, and others [1]) may continue to interbreed when in sympatry [22]. Unexpectedly, because of the considerable evolutionary time (~2.8 million years ago) that separates the roof rat and Norway rat, we observe zero fixed differences (divergent sites) between the two species if published SNP data by Ref. [7] are used (Additional file 1). This implies that the two species cannot be distinguished based on their *vkorc1 *coding sequences. Below we examine whether some coding sequence SNPs published for the Norway rat in fact might belong to the roof rat.

#### Genetic variation in the *vkorc1 *of roof rats from Uganda

**Figure 2:**
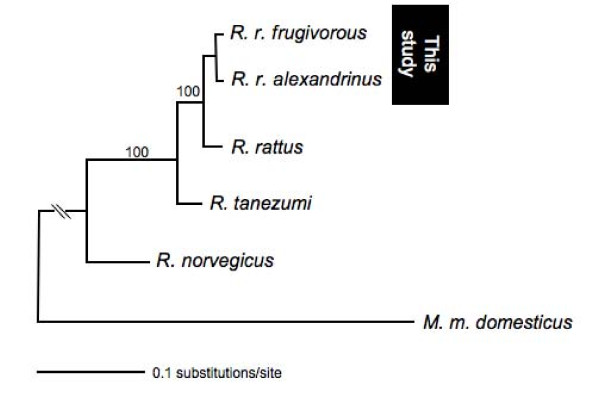
**DNA barcoding for species verification**. MtDNA haplotype tree based on the *CO1 *mtDNA gene confirming our samples are *R. rattus*.

*R. r. alexandrinus *and *R. r. frugivorus *trapped at our study sites in Uganda, Africa (see Methods) are not genetically distinguishable at the level of the *vkorc1 *coding sequence (Additional file 1) and non-coding sequences (Additional file 2). We identified at least 5 *vkorc1 *genotypes (H1-5), but none of these was specific to one or the other nominal subspecies. DNA barcoding based on the *cytochrome oxidase 1 *(*CO1*) mtDNA gene (DNA barcoding) sequences confirmed that our samples are *R. rattus *(Figure 2).

**Figure 3:**
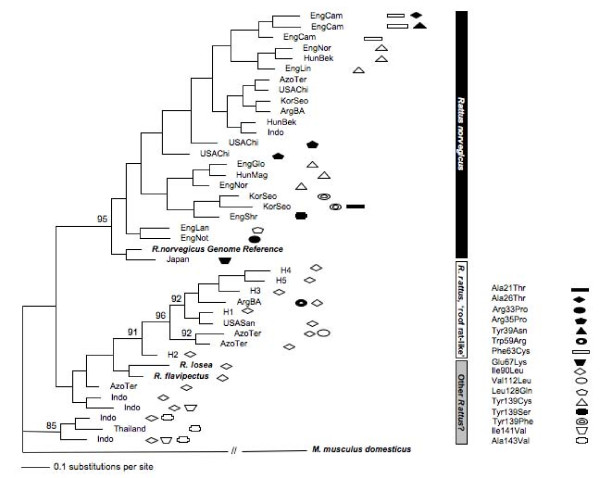
**Genealogies of *vkorc1 *protein coding DNA sequences determined by phylogenetic analysis**. Bootstrap support >90% for clades is indicated at nodes. Samples are labeled as in Additional file 1. Non-synonymous mutations identified in population samples are shown as symbols (see legend). H1-H5 denote roof rat sequences obtained in our study and verified as roof rats by DNA barcoding (Figure 2). Species designations inferred from *vkorc1 *sequence analysis are provided as vertical bars (right panel), but we note that species designations for other *Rattus *species (e.g. samples from the Azores/Terceira, Indonesia, and Thailand) are inconclusive. Note that recombination in the data should be assumed was not considered during phylogenetic analysis. For example, the separate clustering of mutations at position 139 does not imply that mutations at this position have evolved independently. Separate clusters could have originated by recombination.

No barriers to gene flow between these morphs (a.k.a. subspecies) were supported. First, genetic differentiation (as measured as F_ST_) was non-significantly different from zero. Second, sequences H1-5 clustered together during phylogenetic analysis (Figure 3). Thus we predict that if any *vkorc1*-mediated resistance were detected in one of the color morphs or nominal subspecies, interbreeding would transmit it between the two morphs in our study area in Uganda. The absence of non-synonymous polymorphisms in the individuals/populations studied by us so far suggests that resistance does not segregate as a *vkorc1*-mediated trait at present in the study area in Uganda, or that it is too rare to be detected. Among three silent genetic polymorphisms in exon sequences of the roof rat, a synonymous SNP (Gly60Gly, Additional file 1) has not been documented before in rodents. The lack of differentiation at *vkorc1 *between subspecies lends support to those who argue against the legitimacy of subspecies classifications in roof rats based on coat color, which was shown to segregate within crosses between subspecies [23].

### The value of the roof rat v*korc1 *sequence as a reference and outgroup

#### Detection of SNPs of uncertain species origin

Tabulation of sequence differences between sequences of the Norway rat and roof rat revealed polymorphism to divergence ratios for synonymous and non-synonymous sites that deviate from expectations (Table 1). Specifically, only 10 and 14 polymorphic synonymous and non-synonymous SNPs, respectively, were observed in the original data on SNP variation in the Norway rat [7]. Synonymous and non-synonymous divergent sites, however, were not observed (Additional file 1).

**Table 1:** Polymorphism and divergence in *vkorc1 *from Norway rats (MacDonald-Kreitman table).

	Synonymous sites	Non-synonymous sites	Total
***R. rattus *H1-H5 and original *R. norvegicus *data from **[**7**]			

Divergence	0	0	0

Polymorphism	10	14	24

Total	10	14	24

			p-value - undefined

***R. rattus *H1-H5 and *R. norvegicus *data from **[**7**]**after removal of *vkorc1 *SNPs likely to be from roof rats or of uncertain species origin**			

Divergence	4	1	5

Polymorphism	5	9	14

Total	9	10	19

Fisher's exact test, 1-tailed, p-value = 0.119; 2-tailed, p-value = 0.141			

***R. rattus *H1-H5 and *R. norvegicus *data from **[**7**]**after removal of *vkorc1 *SNPs likely to be from roof rats or of uncertain species origin and after removal of singletons**			

Divergence	4	1	5

Polymorphism	5	6	11

Total	9	7	16

Fisher's exact test, 1-tailed, p-value = 0.231; 2-tailed, p-value = 0.308			

One or two sites were divergent between the roof rat and *R. flavipectus *or *R. losea*, respectively. The lack of divergence between the roof rat and Norway rat thus is unexpected, given that the two species have diverged much longer ago (~6 times) than the species triad composed of roof rat, *R. flavipectus *and *R. losea*. This observation is also unexpected in terms of evolutionary rate calculations. Assuming ~2.2 × 10^-9 ^substitutions per site we would expect ~3 divergent sites in *vkorc1 *protein coding region between roof rat and Norway rat.

In addition to the unexpected lack of divergence, we observed peculiar patterns of SNP sharing between samples analyzed previously [7] and our newly obtained roof rat sequences (Additional file 1). For instance, the reported genotypes derived from a series of amino acid substitutions in the USA (Santa Cruz), Argentina (Buenos Aires) and the Azores (Terceira) corresponded to those of the roof rat, not the Norway rat (Additional file 1).

To investigate this issue in more detail, we reconstructed genealogies for the *vkorc1 *coding regions from the roof rat, *R. flavipectus*, *R. losea*, and Norway rat sequences (Figure 3). We observed that 23 sequences obtained from 95 samples grouped with the Norway rat reference sequence (bootstrap value 95%). In contrast, 10 *vkorc1 *sequences obtained from 29 samples, all presumed to be Norway rats, did not cluster with the reference sequence of the Norway rat obtained from Ensembl or Genbank (Additional file 1). Instead, four of these grouped significantly with our newly obtained roof rat sequences H1-H5 (bootstrap value 91%), or grouped loosely together outside the clades of roof rat and Norway rat.

We noted the presence of similar such sequences resembling the roof rat or roof-rat like *vkorc1 *in a study conducted in France [24], 'designated as 'haplotypes C and D' in that study. Therefore, *vkorc1 *SNPs as reported in Ref. [7] obtained from rats in the USA, Argentina and the Azores appear to be *vkorc1 *SNPs from roof rats mistakenly assigned as SNPs from the Norway rat, and other SNPs presumed to be of Norway rat origin from the Azores, Indonesia, Thailand and, possibly France (as reported in Ref. [24]), may be of roof rat origin and/or of uncertain species origin in need of verification. Such sequences could either be divergent lineages of the roof rat, which is paraphyletic with respect to other species of the *Rattus*-species complex [19], or could be other species of *Rattus *that occupy an intermediate position in evolutionary/phylogenetic terms (c.f. [19] for possible such species). However, we are confident stating that those sequences that co-cluster outside the *R. norvegicus *clade in Figure 3 are, in fact, not Norway rats as claimed, and that sequences that cluster together with our *R. rattus *sequences indeed seem to be roof rats. Again, uncertainties remain regarding the species origin of 'roof rat-like' sequences. DNA barcoding of these samples would be needed to determine the species (for DNA barcoding protocols applicable to rats refer to [19]).

Notably, roof rats emerged as a genetically heterogeneous group of *R. rattus *lineages [25]. Some samples from Asia might be common pest species in this region. These would be species that are closely related to the roof rat or considered roof rats, depending on the taxonomy employed [1,25], such as *R. argiventer, R. tiomanicus*, or *R. sikkimensis*. We suggest that the patterns we observed in Figure 3 (c.f. also Additional file 1) can best be explained by misclassification of samples collected in the field. The roof rat and Norway rat do not produce viable offspring when crossed [26], thus excluding the possibility that the pattern can be explained by hybridization. Cross-contamination between DNA samples and/or PCR reactions in the laboratory is a remote yet possible risk factor to studies.

We reassessed the patterns of polymorphisms and divergence between the Norway rat and roof rat (Table 1) while accounting for the presence of SNPs of questionable (i.e. possible *R. rattus *or *R. rattus*-like) species origin in the published data [7]. Questionable origin of SNPs was judged based on the results of phylogenetic analysis (Figure 3). Their removal from the data set resulted in polymorphism/divergence ratios that are consistent with expectations (Table 1). Note that the number of divergent synonymous sites (n = 4; Table 1, middle) now is consistent with the expected approximate divergence between roof rat and Norway rat (~3 sites in the *vkorc1 *gene, see above). We conducted one-tailed tests to assay for excess polymorphisms specifically. Two-tailed tests were also non-significant. This result indicates further that there is no evidence that *vkorc1 *has evolved under selection (Table 1).

The presence of singleton polymorphisms in datasets can render the Macdonald-Kreitman test insensitive to selection. These singletons artificially inflate the polymorphism category, in that a large proportion is expected to be deleterious and thus not expected to attain higher frequencies and fixation [27]. Moreover, due to the sampling design underlying the discovery of the published *vkorc1 *SNPs (see above) we expected an excess of singleton SNPs in the data. If we account for the presence of such deleterious mutations that segregate at low frequency, then the polymorphism/divergence ratios remain consistent with expectations (Table 1). Thus, based on the analysis of polymorphism to divergence ratios, no evidence for selection on the global sample of Norway rat *vkorc1 *sequences can be established.

Overall, we suggest that published SNP data for the Norway rat require closer inspection. As selection is not apparent in the global sample of *vkorc1 *polymorphisms, evidence for selection is perhaps best sought by studying *vkorc1 *polymorphisms at a local scale, namely within deeply and randomly sampled populations that have experienced selection with warfarin or other anticoagulants. The possibility of species misclassification must be excluded before the population genetic data on the *vkorc1 *gene are interpreted in light of selection with warfarin poison.

Researchers conducting genetic testing often receive only tail clips for analysis. Also non-invasive genotyping [28] as applied to rodent droppings [29] provides larger samples for genetic tests of resistance in rodent populations. Thus the risk of species misclassification should not be underestimated, even though the Norway rat and the roof rat are quite different in appearance. The availability of the roof rat *vkorc1 *sequence will further aid in detecting misclassification errors. If in doubt, i.e. when studying populations where more than one rat species occurs, then we encourage DNA barcoding as well as the use of non-coding *vkorc1 *sequences to obtain more informative DNA nucleotide positions for species assignment.

#### Tests for selection at the local scale

One important hypothesis surrounding *vkorc1 *sequence polymorphisms in rodents posits a role for selection with anticoagulant rodenticides such as warfarin [7,10,11,17,30,31]. In particular, tests for selection on specific mutations that occur in a population may provide further support for their roles in mediating anticoagulant resistance. In fact, warfarin selection and its influence on polymorphisms at the warfarin resistance locus have attained high profile status as an exemplary case for rapid microevolution in a mammal [32,33].

Selective sweeps have been documented in warfarin-resistant populations of rats in Germany [16,17]. In an attempt to recapitulate the results for the published data (Table 1 in Ref. [7]) we analyzed *vkorc1 *polymorphism data. After removing SNPs of questionable species origin (i.e., excluding *R. rattus *and other roof rat-like sequences; see Figure 3 and Additional file 1) only the samples from England, Hungary, and Korea appear suitable for formal analysis. The population from Chicago, USA is a strain maintained in a laboratory, which is not suitable for analysis.

We found no statistical support for a recent selective sweep in the population from Korea (Tyr139Phe). More extensive sampling might increase the power for the analysis of this population. Moreover, we found no evidence for a selective sweep in the combined sample from England. However, the sample from Cambridge/Essex in England (Phe63Cys), if analyzed separately, emerged as significant (Tajima's D = -1.84; p < 0.001; Fay & Wu's normalized H = -3.27, p = 0.007).

**Figure 4:**
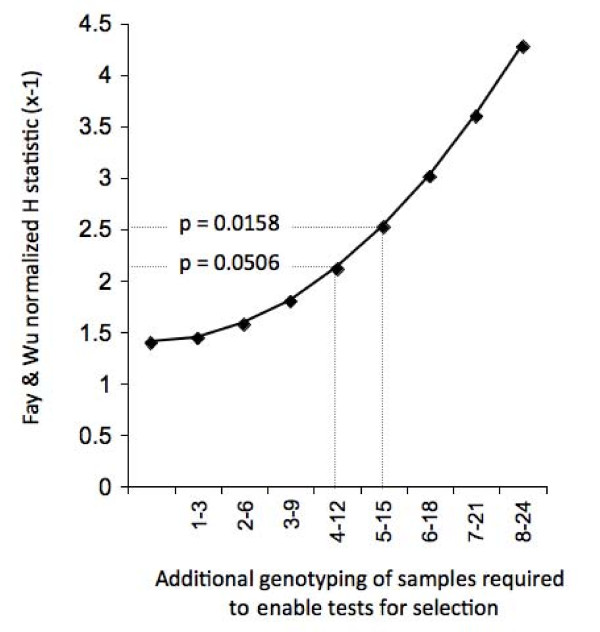
**Simulation of the number of additional genotypes required for detecting selection on *vkorc1 *in the population from Hungary using the Fay and Wu normalized H statistic**. Ref. [7] obtained synonymous variation for 3 of 12 rats available for that study. Ile82Ile was detected in 3/3 of those rats. The non-synonymous polymorphism at high frequency is Tyr139Cys, occurring in 10/12 rats. We simulate the number of additional rats with Ile82Ile that need to be identified in the nine remaining unanalyzed rats to yield significance for a selective sweep in this population. Significance levels 0.05 and 0.01 are indicated.

Finally, an exploratory analysis of the population from Hungary (Tyr139Cys) is used here to illustrate the difficulties in conducting rigorous tests for selection based on *vkorc1 *SNP data as commonly collected and reported (e.g., Refs. [7,24]). Specifically, for a portion of the rats in the Hungarian sample, synonymous SNPs were not assayed (9/12 rats; [7]). We used the information that three out of three rats assayed for synonymous SNPs carried an Ile82Ile synonymous SNP. Amongst these was one rat with the Tyr139Cys variant. In Figure 4 we plot Fay and Wu's normalized H versus additional genotyping efforts needed to obtain sufficient synonymous variation data to enable tests for the signature of a selective sweep at *vkorc1*. We show that a total of seven rats would need to carry the derived Ile82Ile synonymous variant to obtain marginal significance for Fay and Wu's H statistic. Genotyping of at least an additional 4-12 rats for synonymous variation likely would have been sufficient to detect these, and thus, to document a selective sweep in the population (Fay and Wu's normalized H = -2.112, p = 0.0506; Figure 4).

In summary, it appears that the SNP variation data for *vkorc1 *in a number of populations are indicative of selection at the local (population) scale. The high frequency attained by a number of non-synonymous SNPs (e.g., aa position 139; c.f. Additional file 1) is further indicative of a selective advantage for particular SNPs [7,24]. Moreover, if interpreted in conjunction with *in vitro *studies showing VKOR activity in the presence of rodenticide, a selective advantage of rats carrying particular SNPs in *vkorc1 *can be hypothesized. However, to firmly establish selection on particular *vkorc1 *mutations we suggest that future studies need to consider larger numbers of rats collected at random from local populations. Moreover, both non-synonymous and synonymous/intronic SNPs should be assayed to enable tests for genetic hitchhiking. Species misclassification must be ruled out before analyzing and interpreting SNP data.

## Conclusion

We have developed a PCR-based strategy to obtain genomic sequence of the *vkorc1 *gene from the roof rat (*R. rattus*). The primers developed should facilitate isolation of the corresponding region in most rat species because our approach worked equally well for the Norway rat, one of the most divergent *Rattus *species. In addition to the value of having the roof rat *vkorc1 *sequence available for the design of genetic testing studies, we report examples that illustrate the value of this sequence for the interpretation of SNPs that have been published for the Norway rat. Inclusion of the roof rat sequence as a reference and outgroup enabled the detection of SNPs in *vkorc1 *that may not be of Norway rat origin but rather from roof rats or other *Rattus *species.

The outgroup perspective provided by the roof rat sequence allowed for exploration of selective neutrality for *vkorc1 *polymorphisms. The SNP-based evidence accumulated so far is insufficient to conclude with certainty that selection has acted upon any particular SNP in the gene even though previous work based on microsatellite data had shown that the classical case of selection for the resistance phenotype in rats can be detected at the genomic position where the resistance gene (i.e. *vkorc1*) has been mapped [16,17]. The identification of the *vkorc1 *as the molecular target for anticoagulants [5-7,11] now should enable the design of studies to verify selection at the resolution of individual nucleotide sites, but such studies need to be designed such to enable the application of rigorous test for natural selection.

## Methods

### Sampling

Animal material (tails stored in 70% ethanol) was collected from five small villages located within an ~30 km radius in the area bordering the Arua and Nebbi Districts, located ~300 km northwest of Kampala, Uganda, by the Centers for Disease Control and Prevention, Division of Vector-Borne Infectious Diseases (Fort Collins, CO, USA). We obtained nine rats (four *R. rattus alexandrinus *and five *R. r. frugivorus*) from Jupakonja, nine rats from Mawa/Angenja (5 *R. r. al*., 4 *R. r. fr*.), six rats from Oyaragada (3 *R. r. al*., 3 *R. r. fr*.), one *R. r. fr*. from Paganza and one *R. r. al*. from Anyiku Villages. All animal-handling procedures were performed according to protocol ACUC #07-014 under which the field research is conducted as approved by the Centers for Disease Control and Prevention, Division of Vector-Borne Diseases.

Two nominal subspecies of roof rat that are distinguished by their coat color were represented in our sample: *R. rattus alexandrinus *(*R. r. al*.; n = 13) with a brown back and grey belly and *R. rattus frugivorus *(*R. r. fr*.; n = 13) with a brown back and cream belly. While subspecies status based on coat coloration in roof rats has been questioned [23,34], we nonetheless considered the presence of the two color morphs for two reasons. First, coat color variation is evidence for genetic variation segregating in the population; therefore, by collected both morphs we minimized the possibility of collecting an inbred subset of the population with no expected SNPs in the *vkorc1 *gene. Second, we investigated the possibility that color morphs might be genetically differentiated with respect to *vkorc1*-mediated tolerance.

### **DNA extraction and amplification**

Genomic DNA was extracted using a DNeasy^® ^Tissue Kit (Qiagen, Hilden, Germany) following the manufacturer's instructions. We observed that PCR from genomic DNA isolated from tails often was of low yield or failed, which was due to high concentrations of collagen in the DNA. Samples worked satisfactorily by increasing the volume of MgCl_2 _(to 3.3 μl) in the PCR mix and removing the skin prior to DNA extraction. For initial PCR amplification of the *vkorc1 *genomic region, five pairs of primers were designed. Primer design was based on an alignment of 1000 bp upstream and downstream of the *vkorc1 *gene from *R. norvegicus *(GenBank accession no. NC_005100) and *M. musculus *(NC_000073). Forward (F) and reverse (R) primers are as follows: VK1-F (5'-TGC AGC CTC TCC AAC TAC AAT-3'), VK1-R (5'-ATG TGC CAC CTC ACA AAC AA-3'), VK2-F (5'-GCC CTC TCA CTG TAC GCA CT-3'), VK2-R (5'-TGG TGG AAG TGA AAG AAS CA-3'), VK3-F (5'-GAG CCM TCK CAC CAA CTC CT-3'), VK3-R (5'-CAA CCT GGA ATG TCT CC-3'), VK4-F (5'-CTT TAG GTT TAG GTA GCC AGA GG-3'), VK4-R (5'-CYA AGG CAA AGC AAG TYA KG-3'), VK5-F (5'-TTK GGA GAC ATT CCA GGT TGA-3'), VK5-R (5'-GTG YCA GGT TTT GCT GAC TA-3'). Using this set of primers we obtained sufficient sequence data to build contiguous consensus sequences of *vkorc1 *from *R. rattus*. However, the process was inefficient in terms of amplification and tedious in terms of the number of amplicons and sequencing reactions required to cover the genomic region.

The alignment of *R. rattus *(our data) and *R. norvegicus *enabled the design of improved primers that more effectively and economically amplify the entire *vkorc1 *genomic region of *R. rattus *and *R. norvegicus *in three overlapping segments (Rr1-F/R, Rr2-F/R and Rr3-F/R primers; Figure 1).

PCR cycling conditions for primer pairs Rr1-F/R, Rr2-F/R and Rr3-F/R were as follows: 94°C for 2 min 30 s, 31 cycles of 94°C for 30 s, 54°C for 30 s, 72°C for 1 min, followed by an extension time of 72°C for 5 min, and idling at 4°C for storage until processing. Each PCR mixture contained 3 μl 5× Green GoTaq^® ^(Promega, Madison WI, USA), 0.5 μl of each 10 mM stock of F and R primer, 0.3 μl dNTP (10 mM), 0.07 μl Taq polymerase (Promega, Madison WI, USA), 10 μl sterile deionized water, 1.8 μl DNA and 1.8 μl MgCl_2 _(25 mM). PCR samples were cleaned using ExoSAP-IT^® ^(USB Corporation, Cleveland, OH, USA) prior to sequencing on an ABI Prism genetic analyzer sequencer (Applied Biosystems, Foster City, CA, USA).

To confirm that our rat samples are roof rats (*R. rattus*) we obtained sequences of the *COI *mtDNA gene (DNA barcoding) for one sample of each nominal subspecies. We followed the DNA barcoding protocol as described [25]. *CO1 *sequence information for *R. rattus *(EF186585), Norway rat (NC_001665), *R. tanezumi *(EF186623) and the mouse (*M. m. domesticus; *NC_006914) were retrieved from GenBank.

### Analyses

Sequences were assembled using the software package LASERGENE 7.2 [35] and SNPs were confirmed by manual inspection. Finalized sequence alignments were generated using the Muscle algorithm [36] at standard settings as implemented in the software package SEAVIEW 4.2. [37]. Sequences are deposited with GenBank http://www.ncbi.nlm.nih.gov/ under accession numbers HM181979-HM181985 (*vkorc1*) and HM181986-HM181987 (*COI*).

Protein sequence information for *R. norvegicus *(NM_203335), *R. losea *(EF028346), *R. flavipectus *(FJ868832), *R. rattus *(this study) and the house mouse (*M. musculus*; BC031732) were retrieved from GenBank. In addition, SNP data for 124 rats (presumed to be Norway rats; see Results) were downloaded (c.f. Table 1 for SNP accession numbers) and assembled into sequences assuming that data published in [7] represent haplotypes/homozygous genotypes. Unfortunately no information (e.g., heterozygosity of SNPs) was provided to enable better-informed inferences from published sequence data.

Gene trees were reconstructed for the *vkorc1 *coding region and *CO1 *using the Bayesian method as implemented in MrBayes 3.1.2. The Bayesian inference utilized Markov Chain Monte Carlo (MCMC) simulations in combination with the best Model (HKY+G) [38] in MODELTEST 3.7 [39]). Four chains were set to run simultaneously for 1,000,000 generations during the MCMC process, with trees being sampled every 100 generations. After eliminating the first 1,000 trees as burn-ins, a majority-rule consensus tree was constructed with nodal values representing the posterior probability.

Population genetic analyses were conducted with the software package DnaSP v5. [40] and Arlequin 3.0 [41]. These included analysis of genetic differentiation/gene flow calculated as F_ST_, the MacDonald-Kreitman test for selection and basic and composite measures of polymorphism data (θ, π, Tajima's D). If an outgroup perspective was needed the Norway rat was used to polarize mutational change in the roof rat, and the roof rat was used to polarize mutational changes in the Norway rat. As we describe in the Results section, we used the findings from the phylogenetic analysis to detect contamination of the *vkorc1 *SNP dataset for the Norway rat with SNPs that must originate from roof rats.

The Fay and Wu test [42],; http://www.genetics.wustl.edu/jflab/htest.html, accessed March 2010] was performed with 5000 coalescent simulations, assuming no back mutation and recombination. Under these parameter settings the test is particularly sensitive to detect selection. We opted for these parameter settings because the data are too limited to apply less sensitive and more conservative parameter settings at this time. We suggest that future studies testing for selection on more comprehensive samples should apply more conservative parameter settings, namely, they should consider recombination.

## Competing interests

The authors declare that they have no competing interests.

## Authors' contributions

MHK conceived and directed the study and wrote the manuscript with help of the co-authors. JCD and AM conducted the laboratory work and analyses were done with the help of YS. JNB conducted the fieldwork and handled most of the sampling logistics. All the authors approved the final manuscript.

## Supplementary Material

Additional file 1 **DNA nucleotide variation in the *vkorc1 *protein coding region**. Shown are variable (polymorphic/divergent) amino acid positions (aa position) and variable nucleotide positions (nt position provided relative to the start codon) for the coding region (E1-E3) of *vkorc1*. Non-synonymous mutations are labeled as nucleotide change followed by resulting amino acid change. We provide the consensus amino acid and DNA sequences for the roof rat, as deduced from five genotypes (H1-H5) obtained by sequencing the specimens assigned, based on coat color, as two nominal subspecies *R. rattus alexandrinus *or *R. r. frugivorus*. The sequences of *R. flavipectus *and *R. losea *are provided. Note three polymorphisms in *R. losea *(Ser35Cys, Ser58Gly, Cys96Cys) are not depicted. Sequences reconstructed from SNP data for the published *vkorc1 *coding sequence [7] are shown as part of a group we call *R. rattus*/other *Rattus and R. rattus*-like and as *R. norvegicus*. The published sequence from *R. norvegicus*, as retrieved from GenBank and Ensembl, was used to aid identification of 'genuine' *R. norvegicus *sequences/SNPs. These groupings were defined based on phylogenetic analysis (Figure 3). The mouse sequence (*M. musculus domesticus*) is provided for reference. The bottom panel (ss#) lists the SNP accession numbers [7]. Note the Ser56Pro SNP was not published (Table 1 in [7]) but was reported as a SNP in *R. norvegicus *without referring to the source population analyzed. A SNP underlying the Leu120Gln variant has been published [11], but for a population not included in the previous study [7]. Sample origins are denoted as follows [7]: USA/Santa Cruz (USASan, n = 3), USA/Chicago (USAChi, n = 18) Argentina/Buenos Aires (ArgBA, n = 15), Azores/Terceira (AzoTer n = 5), Indonesia (Indo, n = 16), Thailand (Thailand, n = 2) England Cambridege/Essex (EngCam, n = 17), England/Norfolk (EngNor, n = 2), England/Gloucestershire (EngGlou, n = 1), England/Lincolnshire (EngLin, n = 2), England/Shropshire (EngShr, n = 2), England/Nottinghamshire (EngNot, n = 2), Hungary/Bekes (HunBek, n = 3), Hungary/Maglod (HunMag, n = 9), Korea/Seoul (KorSeo, n = 8), and Japan (Japan, n = 6). Click here for file

Additional file 2 Non-coding sequence data for the *vkorc1 *of the roof rat (new data) and published data on other rat species. Click here for file
